# Evaluation of the feasibility of acetabular cup pre-determination in revision total hip arthroplasty via X-ray of the bone stock of the anterosuperior acetabulum

**DOI:** 10.1186/s13018-021-02745-3

**Published:** 2021-10-14

**Authors:** Jingwei Zhang, Keyu Kong, Yingjun Chi, Xiaoliang Liu, Yiming Zeng, Huiwu Li

**Affiliations:** 1 Shanghai Key Laboratory of Orthopaedic Implants, Department of Orthopaedic Surgery, Shanghai Ninth People’s Hospital, Shanghai Jiaotong University School of Medicine, 639 Zhizaoju Road, Shanghai, 200011 People’s Republic of China; 2 Department of Orthopedics, Shengzhou People’s Hospital (the First Affiliated Hospital of Zhejiang University Shengzhou Branch), No.666, Dangui Road, Shengzhou, 312400 Zhejiang People’s Republic of China

**Keywords:** Bone defect assessment, X-ray, Revision hip arthroplasty, Prosthesis selection

## Abstract

**Purpose:**

This study was aimed to explore (1) location on AP pelvic X-ray that displayed bone stock in anterosuperior acetabulum; (2) whether X-ray could provide enough evidence to evaluate whether bone stock could provide support for acetabular cup; (3) criteria to determine whether anterosuperior bone stock could provide sufficient support for cup on X-ray.

**Methods:**

Our study retrospectively collected 43 patients who underwent revision THA for cup loosening from 2014 to 2019. The position of anterosuperior acetabular bone stock was compared between X-ray and CT-based 3-D reconstruction. Seventy-millimeter acetabular cup was implanted simulatively to obtain the contact line between acetabular cup and superolateral remaining bone stock. The contact line length and the angle were measured. Patients were divided into cup group and cage group, and ROC curves of both contact line length and angle were drawn.

**Results:**

The superolateral part of acetabulum on X-ray could reflect the anterosuperior host bone stock of acetabulum according to the comparison of anteroposterior pelvic X-ray and 3-D reconstruction. Critical point was chosen when we got the highest sensitivity with a 100% specificity in ROC curves. The critical values of contact length and angle were 15.58 mm and 25.5°.

**Conclusions:**

Surgeons could assess the anterosuperior bone stock of acetabulum by AP pelvic X-ray to decide whether revision could be done merely using cup or need customized cage. Clinically, when contact line length was larger than 16 mm or contact angle was larger than 25.5°, adoption of cup could obtain primary stability in the revision surgery in most cases.

## Introduction

Currently, the number of patients undergoing revision total hip arthroplasty (RTHA) is rapidly increasing [1, 2]. Among those who undergo RTHA, bone defects, especially acetabular bone defects, are some of the most common but difficult problems that surgeons face [3–6]. Thus, assessment of acetabular bone defects is critical for surgical planning and preoperative preparation [7].

Pelvic anteroposterior radiography is essential for patients undergoing revision THA. In particular, the evaluation of acetabular bone defects and the host bone stock remains meaningful. Previously, there have been several reports regarding acetabular bone defect assessment and classification, such as the Paprosky classification [8] and AAOS classification [9]. However, similar studies have reported that these classifications remain unsatisfactory in terms of accuracy and reliability [10–16]. Hence, significant errors may occur when different surgeons use these methods. Moreover, this may have adverse effects on preoperative planning [12, 17]. One possible reason for this is the difficulty in using a plain two-dimensional X-ray to show the complex three-dimensional configuration and structure of the pelvis. Therefore, *Choplin* et al. [6] and *Saleh* et al. [17] proposed that anteroposterior (AP) pelvic X-rays could only provide a preliminary evaluation for RTHA; consequently, complex cases required further evaluation such as computed tomography (CT) and CT-generated three-dimensional models [18, 19]. Nevertheless, the role of primary assessment, the precise information that surgeons need to obtain from X-ray, and the significance of X-ray results in pre-surgical planning are still unknown.

In RTHA, deciding whether using a cup alone can provide primary stability is critical in preoperative planning [13, 20, 21]. For surgeons, this may affect the surgical preparation and judgment of the difficulty of the surgery. Our previous study and clinical experience showed that the primary stability of the cup was achieved when the three-point support was distributed over the semicircle around the acetabulum. *Mohamed* et al. [13] introduced a new classification system for acetabular bone defects based on the three-point fixation theory. However, the precise locations of these three points are not illustrated in detail [13]. The rami ischii, rami ossis pubis, and the anterosuperior part of the acetabulum are anatomical structures that have the most bone stock and are most likely to have residual host bone left when bone defects occur around the acetabulum. If the host bone left in these three areas provides sufficient support for the cup, then the acetabular cup is sufficient on the acetabular side. According to our clinical experience, assessing rami ischii and rami ossis pubis based on AP pelvic radiography is relatively easy. The main problem is knowing whether the anterosuperior part of the acetabulum has enough host bone to support the cup, since this is not easily examined in AP pelvic radiography.

This retrospective study aimed to explore the following: (1) whether AP pelvic X-ray could be utilized to display host bone stock at the anterosuperior part of the acetabulum; (2) whether X-rays could provide sufficient evidence to evaluate whether the host bone stock at the anterosuperior part of the acetabulum could provide support for the cup; and (3) the criteria for determining whether the anterosuperior bone stock of the acetabulum could provide sufficient support for the cup based on the AP pelvic X-ray results.

## Methods

### Study participants

This retrospective study was approved by the ethics committee of our institution. All patients who underwent RTHA for cup loosening between January 2014 and December 2019 were included in the study. Patients who refused to be involved in this study were excluded, whereas patients who underwent revision surgery due to infections and loosening of the femoral prosthesis that did not involve acetabular bone defects were also excluded. After the patients visited the hospital, radiographic evaluations of the pelvic and axial views of the affected hip joint were performed. Patients with mild bone defects (Paprosky [8]) were excluded. Finally, 43 patients with obvious acetabular bone defects were included in the study.

### Radiographic evaluation

**Fig. 1:**
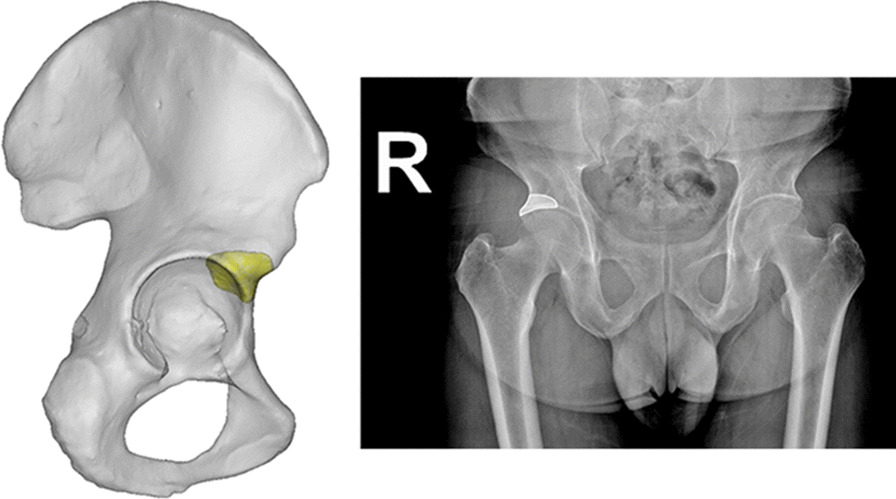
X-ray of the anterosuperior bone stock of the acetabulum

According to the selection of intraoperative prosthesis, the patients were divided into a cup group and a cage group. The cup group included patients who used the cup alone for revision, regardless of whether augment components were used. However, patients in whom screws were used to fix the augment or buttress plate in order to reconstruct the anterosuperior acetabulum were excluded. The cage group included patients who used cup-cage or customized cage for revision surgery. In the cage group, uncemented cups were unable to achieve sufficient fixation and stability after assessment during surgery. Thus, the cages were used for reconstruction. There were 34 and 9 patients in the cup and cage groups, respectively. Preoperative anteroposterior pelvic X-ray and preoperative hip joint thin-slice CT scan results of all patients in the two groups were collected. Three-dimensional reconstruction was performed based on the hip joint CT scan; thus, the influence of metal artifacts on CT was manually adjusted. Using the Medraw PrintV1 software, we matched the CT-based three-dimensional reconstruction model with the X-ray results. Calculations were made to obtain the two-dimensional projections of acetabular anterosuperior bone stock, which were selected in the three-dimensional model. The anterosuperior bone stock was defined as the area of anterosuperior acetabular bone mass thickening extending from the anterior inferior iliac spine. An example of this is shown in Fig. 1.

Preoperative anteroposterior pelvic radiographs of all patients were obtained. We chose a 70-mm acetabular cup and simulated it in the patient’s pelvis on radiographs. The 70-mm acetabular cup was the largest size we could obtain. If the 70-mm cup still could not fit the bone defect, the bone was unlikely to adopt the use of a cup in revision. The lower edge of the acetabular cup was determined using the lower edge of the acetabulum. Moreover, we simulated the cup with an abduction angle of 45°, which was the case in most patients. In addition, 45° was optimal for cups in RTHA.

**Fig. 2:**
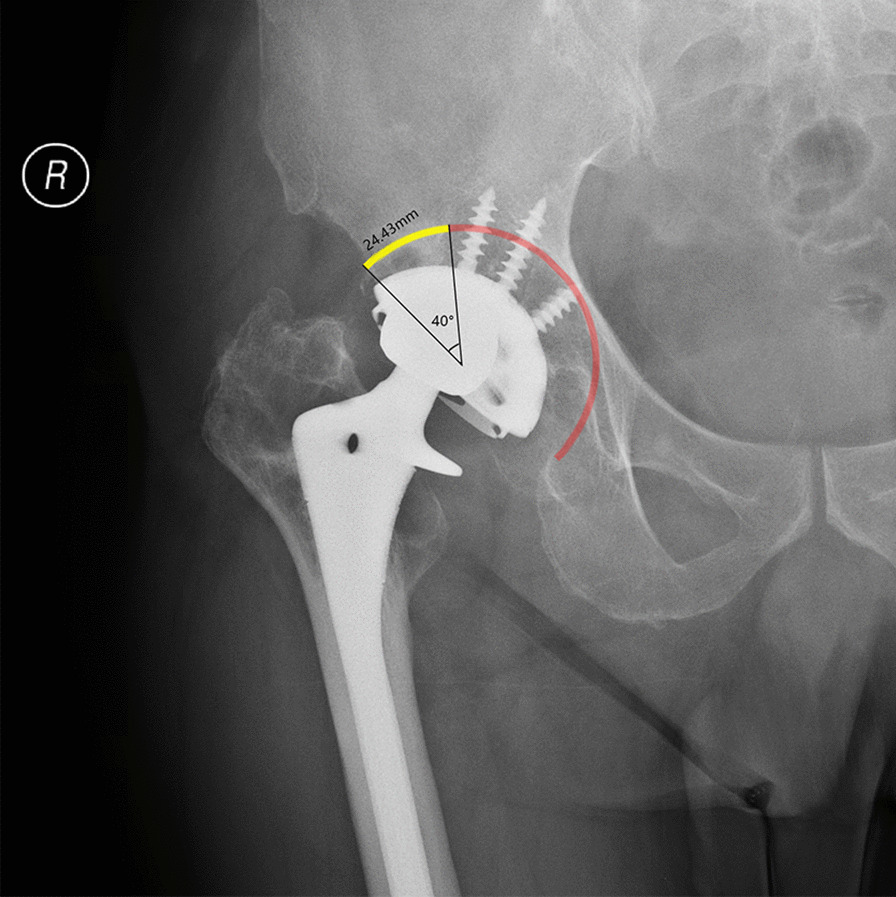
Based on the simulative cup size and position, the contact length and angle between the acetabular cup and the superolateral part of bone stock on the two-dimensional X-ray image were measured

After determining the cup size, we determined the contact line between the cup and anterosuperior bone stock on the X-rays. We then measured the contact length and contact angle between the two contact ends and the rotation center of the hip joint (Fig. 2). To some extent, the contact line indicates the amount of contact between the anterosuperior part of the cup and the host bone, while the contact angle indicates the relative proportion of the anterior superior part of the cup to the host bone.

### Statistical analyses

**Fig. 3:**
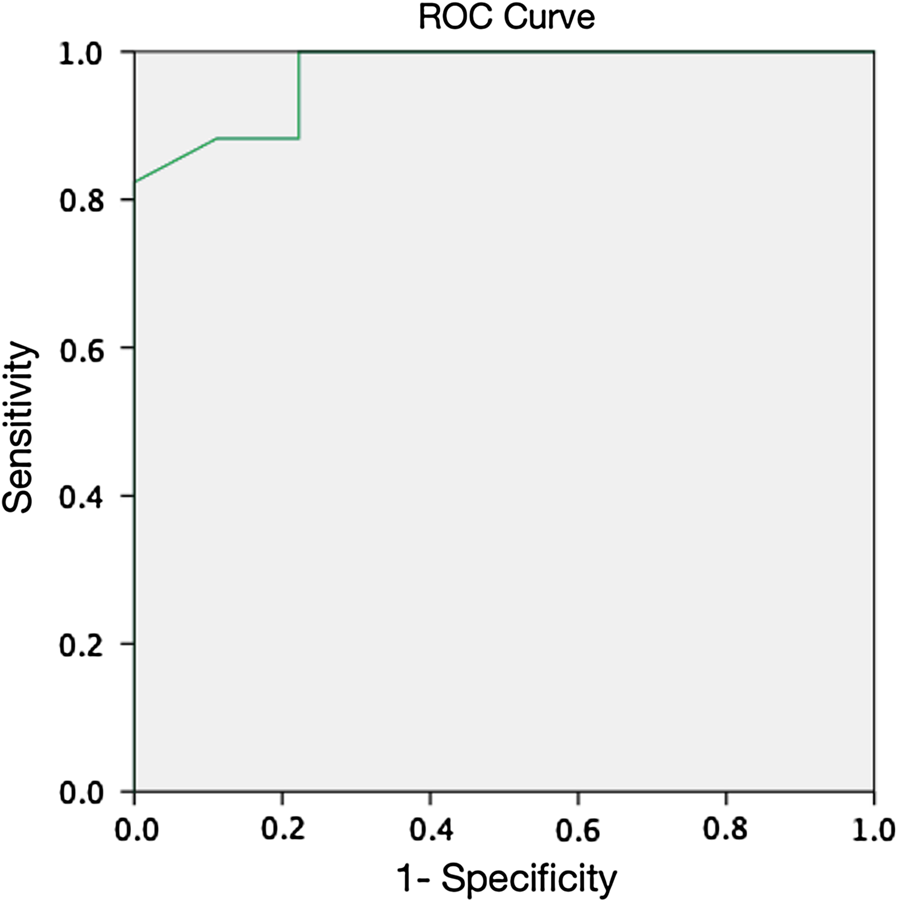
Receiver operating curves (ROC) of the length and the angle of the contact line. The two curves overlap each other

Statistical analyses were conducted using the Statistical Package for the Social Sciences (SPSS) version 17.0, for Windows (Inc., Chicago, IL). Receiver operating curves (ROC) were separately constructed using the length and angle of the contact line between the acetabular cup and anterosuperior bone stock, which were measured via X-rays of the patients in the two groups (Fig. 3). The critical point was chosen when the sensitivity was the highest, with 100% specificity. According to our data, the critical contact length was 15.58 mm, while the critical contact angle was 25.5°.

## Results

The patients’ demographic information and basic information of cups and cages are listed in Table 1, and the cup size of patients in the cup group is listed in Table 2. The anterosuperior host bone stock of the acetabulum in the three-dimensional model is shown as the superolateral part of the acetabulum on the AP pelvic X-ray (Fig. 1).

**Table 1:** Patient demographic background information

Demographic	Cup group	Cage group
*Gender, n (%)*		
Female	22 (64,7%)	6 (66.7%)
Male	12 (35.3%)	3 (33.3%)
Age at surgery, mean (range)	63.78 (47–81)	67.47 (30–92)
Site (left/right)	17/17	4/5
Type of cup/cage	Trabecular Metal Revision Shell (Zimmer Inc., Warsaw, Indiana, USA) (2/34)	Customized cage (9/9)
	Trabecular Metal modular acetabular system (Zimmer Inc., Warsaw, Indiana, USA) (29/34)	
	Pinnacle Gription acetabular cup (Depuy Orthopaedic Inc., Warsaw, Indiana, USA) (3/34)

**Table 2:** Sizes of cups used for patients in cup group

Size of cup	Overall (*n* = 34)
48	1
50	5
52	8
54	1
56	3
58	3
60	3
62	3
64	3
66	2
68	1
70	1

X-ray results could provide surgeons with sufficient information to determine the use of cups based on our comparison of the contact line and the angle between the two groups. The ROC curve showed the critical point at which the highest sensitivity was achieved with 100% specificity. The critical contact angle was 25.5°, while the critical contact length was 15.58 mm. As a result, we concluded that when the contact angle and contact length, measured using the aforementioned measuring method, were over 25.5° and 15.58 mm, respectively, an acetabular cup could be used as a primary revision prosthesis instead of a cup cage or a customized cage. Thus, considering its clinical application, a length of 16 mm is more convenient and practical in clinical practice.

## Discussion

Acetabular bone defects are a major problem in RTHA [3–6, 10]. In revision surgery, assessment of the bone defect via preoperative radiography is critical in deciding whether further evaluations, examinations, or extra preoperative preparations are needed. Moreover, the sufficiency of the cup alone for RTHA and knowing whether a customized prosthesis is needed for surgery are essential for preoperative preparation and surgical difficulty evaluation [5, 6, 10, 22, 23].

In previous studies, there were several reports regarding acetabular bone defect assessment and classification, such as the Paprosky classification [8] and AAOS classification [9]. However, similar studies have shown that their accuracy and reliability are still unsatisfactory when conducting assessments [10–16]. However, it was difficult to assess relatively complex three-dimensional objects using simple two-dimensional images. In addition, the different training levels of doctors made these classifications less repeatable [12]. To assess complex bone defects through these classifications, considerable professional training is required. Currently, there are advanced evaluation methods, such as CT and rapid prototyping, which have been used for surgical planning and prosthesis fabrication for decades [24–28]. However, the need for three-dimensional information on bone defects from AP pelvic radiographs remains unknown. For the above reasons, several scholars believe that X-rays can only provide a rough evaluation [6, 17, 29]. However, the information provided by X-rays and the evaluation that can be made based on X-ray results remain unclear. Nevertheless, this does not mean that the X-rays are not valuable. When performing radiological evaluations of patients who require hip revision, radiography is always the preferred initial examination. Meanwhile, it is very important to perform a primary assessment of the patient through radiography. In particular, for the surgeon, knowing whether the acetabular cup can be used for surgery or whether the cage or a customized prosthesis is needed is essential for preoperative preparation and judgment of the operation difficulty [6, 10, 23, 30, 31]. Therefore, we conducted this study to explore whether X-rays could be used to determine whether patients could use cups for revision surgery.

In revision arthroplasty, we found that the premise for the reliable initial stability of the acetabular cup was whether the cup could acquire a three-point fixation within the boundaries of the acetabular distribution over 180°. The rationale was that only three-point clamping beyond the hemisphere could provide sufficient rotational stability. *Ghanem* et al. [13] also pointed out the importance of three-point fixation when faced with bone defects in revision arthroplasty in their study. However, the study did not specify the detailed requirements of the three-point distribution. Moreover, during the surgery, we found that the rami ischii, rami ossis pubis, and the anterosuperior and posterosuperior parts were the anatomical sites with the most abundant bone stock around the acetabulum. The posterosuperior part is the weight-bearing area of the acetabulum [32, 33]. Therefore, when a severe bone defect occurs around the acetabulum, it is generally the first to be damaged. Furthermore, it is difficult for the posterosuperior part to have an arrangement wherein one point is distributed more than 180° relative to the other two. Therefore, the anterosuperior, rami ischii, and rami ossis pubis are the most likely anatomical sites to form a three-point effective fixation.

The bone stock of the rami ischii and rami ossis pubis is easily distinguished on X-rays, but the exact placement of the anterosuperior part of the acetabulum on X-rays remains unknown. Moreover, there is no standard for determining whether the anterosuperior host bone stock can provide effective support when a bone defect occurs. Therefore, when bone defects occur, it is difficult to assess whether the anterosuperior host bone stock can provide effective support to form a three-point fixation. Therefore, in this study, an AP pelvic X-ray was calculated based on CT-generated three-dimensional models after the cup implantation procedure. We found that the superolateral part of the acetabulum in the AP pelvic X-ray represented the anterosuperior part of the acetabulum in the 3-D model, which was consistent with our experience. This meant that we could assess the condition of the anterosuperior host bone stock of the acetabulum based on the AP pelvic X-ray.

**Fig. 4:**
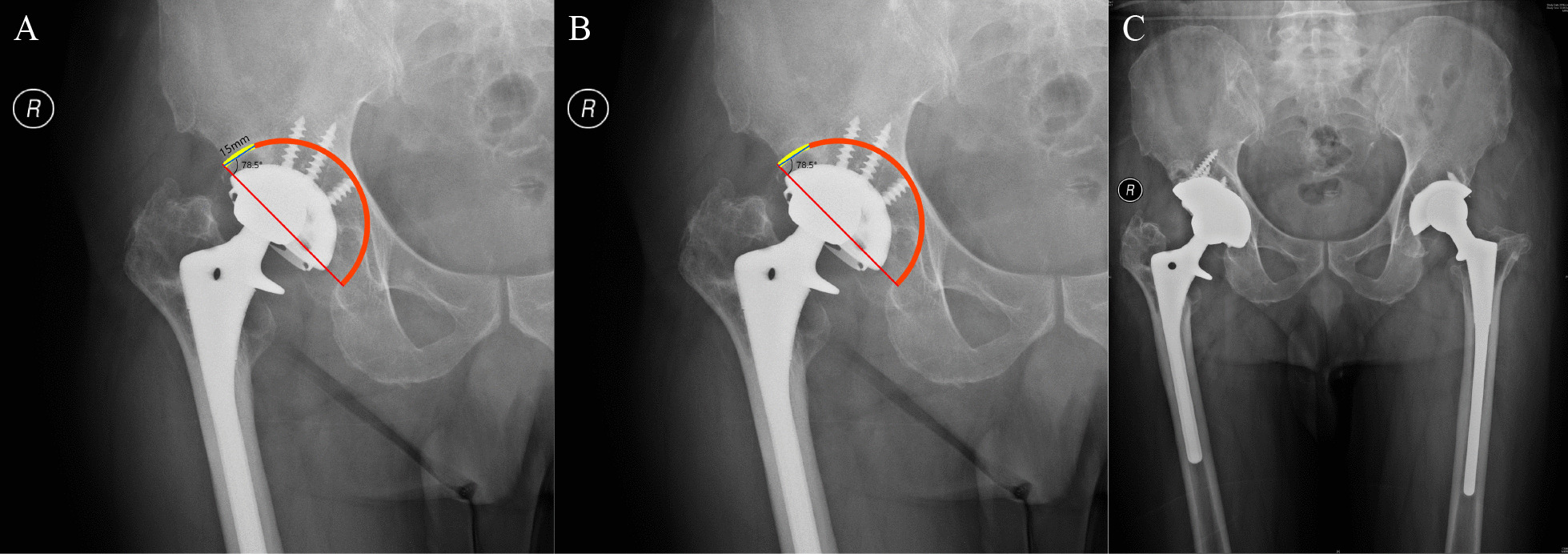
Estimation of the contact length on X-rays in clinical practice. **A** The line with an angle of 78.5° from the simulative diameter can best represent the contact curve. **B** Rough estimation of the contact line length is 15 mm in this patient, which means that he/she is capable of receiving a revision surgery with cup. **C** Postoperative pelvic AP X-rays of this patient

This study also elucidated how to use the superolateral part of the acetabulum on X-ray imaging to determine whether the anterosuperior host bone stock of the acetabulum could provide sufficient support for the cup. In this study, we simulated cup implantation according to X-rays. If the simulated implanted cup and the superolateral part of the acetabulum on X-ray could obtain a certain amount of direct contact, then during the operation, the anterosuperior host bone could provide sufficient support for the cup. The results of the ROC curve indicated that when the contact length was over 15.58 mm or when the contact angle was over 25.5°, the use of an acetabular cup would be sufficient for surgery. When implanting the cup, we first ensured that the lower edge of the cup was in contact with the expected rami ischii and rami ossis pubis. Second, we used an acetabular cup with a diameter of 70 mm, the largest size available, since it could offer stable support for most patients. In clinical practice, surgeons can draw a 70 mm straight line with a 45° angle from the horizontal line between the bottom of the acetabulum and the superolateral part of the acetabulum to simulate the diameter and abduction angle of a 70-mm cup. In this study, a line with a 78.5° angle from the simulated diameter of the cup was drawn, and the distance between the two intersections of this line and the superolateral acetabulum roughly represented the contact line according to our calculation. If the distance was over 16 mm, the single use of the cup was sufficient for revision surgery because the real curved contact line was longer than the linear distance (Fig. 4). In this representative case, anterosuperior bone stock is sufficient to hold the cup with anteroinferior and posteroinferior bone stock. However, posterosuperior bone defect was found in preoperative evaluation and during operation which required an augment to assist the fixation of weight-bearing area.

Our study has several limitations. First, this was a retrospective cohort study; thus, we drew our conclusions after retrospectively comparing the host bone imaging on X-rays between the two groups, which were divided according to different intraoperative selections. However, the surgeon's choice of surgical approach, especially the use of cementless hemispherical/oval cup, cage/cup-cage, or customized prosthesis as the main prosthesis, is somewhat subjective and relies on the surgeon's discretion, which results in inevitable deviations. Therefore, high-quality prospective studies are required to verify the results of this study. Nonetheless, although several patients in the cage group could have used the acetabular cup, all patients in the cup group were verified intraoperatively to be eligible for cup use alone. Our selection method of the critical point guarantees that all patients satisfying the demands of the critical point could receive a revision THA with an acetabular cup. Second, the assessment of bone defects in several patients with RTHA is relatively difficult. If the surgeon suspected that the bone defect was serious, it was recommended to perform further evaluation via CT, three-dimensional reconstructions, or rapid prototyping [24–28]. The X-ray assessment method used in this study was only intended to provide the primary assessment of the bone defect and to offer a reference for the selection of a surgical approach, which enables surgeons, especially doctors with less experience, to easily grasp the reference criterion when using X-rays to conduct a primary assessment of patients. Third, this study did not conduct long-term follow-up of the included patients, and there were no reports on the long-term implant survival rate of revision joints. We plan to continue follow-up with these patients and report the follow-up results in due time. Finally, the number of patients included in this study was limited. Thus, we will attempt to enroll more cases in the future.

## Conclusions

The results of this study show that the superolateral part of the acetabulum on AP pelvic radiographs can reflect the anterosuperior host bone stock of the acetabulum in patients who require RTHA. Based on whether the rami ischii and rami ossis pubis are intact, we can judge whether the acetabular cup can be used for revision surgery on the patient. At present, we propose the clinical criteria suggesting that the anterosuperior host bone stock of the acetabulum can provide sufficient support for the acetabular cup. Preoperative anteroposterior pelvic radiographs showed that the contact length of the simulated implanted cup and the superolateral host bone was greater than 16 mm, and the angle between the lines connecting each end point of the contact line to the rotation center was greater than 25.5°.

## Data Availability

The datasets generated and/or analyzed during the current study are not publicly available because other studies are being carried out based on these data, but are available from the corresponding author upon reasonable request.
